# A review and evaluation of orthodontic brackets, molar bands and orthodontic auxiliaries during orthognathic surgery: A prospective cohort study

**DOI:** 10.1177/14653125231186825

**Published:** 2023-07-18

**Authors:** Romee ME van Ommeren, Tom CT van Riet, Jean-Pierre TF Ho, Ronald EG Jonkman, Alfred G Becking

**Affiliations:** 1 Department of Orthodontics, ACTA Amsterdam, Amsterdam, the Netherlands; 2 Department of Oral and Maxillofacial Surgery, Amsterdam UMC location AMC, Amsterdam, the Netherlands

**Keywords:** orthognathic surgery procedure, orthodontics, failure rate, orthodontic appliance, orthodontic auxiliaries

## Abstract

**Objective:**

The primary aim of this study was to provide a review of the types and frequency of orthodontic brackets, molar bands and orthodontic auxiliaries used for patients undergoing orthognathic surgery. The secondary aim was to evaluate the risk of failure of these items during orthognathic surgery.

**Methods:**

From three Dutch hospitals, 124 adult patients were included in this prospective cohort study. Five independent researchers collected the data during surgery using a specifically created data extraction form. The type of surgery, surgeon, orthodontist and type of orthodontic bracket, molar band or auxiliary were noted for each tooth. To evaluate their failure risk, the following variables were noted: failure and site; and type and cause of failure.

**Results:**

Stainless-steel brackets were the most frequently (75.8%) used bracket type seen in patients undergoing orthognathic surgery. Ceramic brackets were seen in 24.2% of the cases and were only applied in the anterior region. Molar bands were present in 58.9% of the patients and mostly with bands on the first molars in combination with bonded tubes on the second molars. In 32.2% of all cases, one or more failures were noted. One-third of all failures were described as detachment of the molar tube on the most posterior molar. Kobayashi ligatures and powerpins showed the highest risk of failure (odds ratio [OR] 3.70, 95% confidence interval [CI] = 1.91–7.15). No significant difference in failure rate was found between stainless-steel brackets, molar bands (OR 0.34, 95% CI = 0.08–1.43) and ceramic brackets (OR 0.44, 95% CI = 0.14–1.45).

**Conclusion:**

Stainless-steel brackets, ceramic brackets, molar bands and surgical hooks are suitable for orthognathic cases. Kobayashi ligatures and powerpins had a significantly higher risk of failure so are not recommended for temporary intraoperative maxillomandibular fixation (TIO-MMF).

## Introduction

Patients with extensive dentofacial deformities may require combined orthodontic and surgical treatment (Precious et al., 1985). These treatment plans are considered challenging due to the multidisciplinary workflow and necessity of meticulous treatment planning (Gandedkar et al., 2016). The role of the orthodontist in an orthognathic treatment plan is not only essential in the initial diagnostic phase and the pre- and post-operative orthodontic phases, but also during the intra-operative phase.

During orthognathic surgery, occlusal splints are used as a device for placing the mandible or maxilla in the pre-planned position (Irving et al., 1984). With the splint in position, the mandible and maxilla require ‘temporary intra-operative maxillo-mandibular fixation’ (TIO-MMF). TIO-MMF is essential to ensure that the split or cut jaw is kept in the planned position to allow for adequate fixation with surgical plates and/or screws. The TIO-MMF is created by tightly applying stainless-steel wires or, alternatively, power chains to orthodontic fixation points in the upper and lower arches (van Ewijk et al., 2021).

Failure of these orthodontic fixation points during orthognathic surgery can reduce the predictability of the outcome because of malpositioning of the splint and the possibility of fixing the cut or split segments in a suboptimal position. Failure or absence of fixation points might lead to an increase in the duration of the surgery or can cause severe complications due to bracket or auxiliary loss into the surgical wound or airway (de Queiroz et al., 2013; Laureano Filho et al., 2008; Shaeran and Samsudin, 2018).

Despite the relative high frequency of orthognathic surgery, surprisingly little is known about the use of different types of orthodontic brackets, molar bands and auxiliaries as temporary fixation points during the surgical phase, and their risk of failure. A limited number of studies on this subject has focused on the differences in prevalence and failure rates for bonded versus banded molar tube brackets and have been based on postoperative radiographs (Attishia et al., 2015; Godoy et al., 2011; Millett et al., 1999).

As orthodontists and oral and maxillofacial (OMF) surgeons may have preferences on which orthodontic brackets, molar bands and auxiliaries to use during the intra-operative phase, the authors noted a great variation in their presence during surgery. A review of their frequency and failure rate is missing in literature.

The primary aim of this prospective cohort study was to provide a review of the types and frequency of orthodontic brackets, molar bands and orthodontic auxiliaries used during orthognathic surgery in a representative sample of surgical patients in the Netherlands. The secondary aim of this study was to evaluate the risk of failure of these components during the orthognathic surgery.

## Methods

To obtain a representative dataset, a prospective cohort study was designed. Between November 2019 and July 2021, patients undergoing orthognathic surgery at three hospitals in the Netherlands were included. Different OMF surgeons from the Amsterdam University Medical Center (location AMC), Kennemer & Meer and Spaarne Gasthuis (Haarlem) and Northwest Clinics (location Alkmaar) participated in each hospital. Patients were referred by a variety of orthodontists. A request to the Medical Ethics Review Committee (MERC) of Amsterdam UMC was submitted at the start of this study to assess whether the research was subject to the Medical Research Involving Human Subjects Act (WMO). The MERC confirmed that the WMO did not apply to the study and that official approval, from the committee, was not required (reference no. W19_419#19.486, Appendix 2). All patients aged over 18 years and undergoing orthognathic surgery where TIO-MMF was necessary were included, except for the following: patients with orthodontic appliances other than conventional fixed orthodontic appliances, i.e. (clear) aligners; patients where TIO-MMF screws were primarily used as fixation points; patients undergoing orthognathic surgery without the use of TIO-MMF; and patients undergoing surgery with TIO-MMF for purposes other than orthognathic surgery (i.e. trauma surgery).

Data were collected by five orthodontic or OMF surgery residents who were trained to collect data with the use of a specifically created data extraction form (Appendix 1). The data collection form was developed through agreement sessions between the authors and based on previous literature (Godoy et al., 2011; O’Bannon et al., 2006). A list of different types of orthodontic brackets, molar bands and orthodontic auxiliaries was compiled under the supervision of two orthodontists with more than 25 years of experience in their profession. The type of orthognathic surgery, the surgeon, the orthodontist and the type of orthodontic bands, bonds or auxiliary on each tooth were noted preoperatively. Basic properties of the fixed orthodontic brackets and molar bands were noted, such as the material and type of ligation. In addition, the presence of extensions on the brackets, molar bands and other types of orthodontic auxiliaries were noted, such as surgical hooks, Kobayashi ligatures or powerpins.

During the surgery, the OMF surgeon was asked to report any failure of the brackets, bands or auxiliaries and the following variables were noted: site; moment; and type and cause of failure. An appliance was defined as failed if it was no longer usable for TIO-MMF. All included cases were numbered for anonymisation purposes.

All analyses were conducted using SPSS version 27 (IBM Corp., Armonk, NY, USA). Descriptive statistics, such as frequencies and percentage, were computed for each variable. To test the cohesion between the categorical variables, a binary logistic regression analysis was conducted. *P* ⩽ 0.05 was considered statistically significant.

## Results

In this study, 124 patients (63 [51%] men, 61 [49%] women) were included in whom 3228 brackets, 352 molar bands and 2331 possible fixation points were evaluated. Most patients underwent a bilateral sagittal split osteotomy of the mandible (n = 63, 51%) or a bimaxillary osteotomy (n = 46, 37%). Solitary Le Fort I osteotomies were performed in 14 (12%) patients. The patients were referred by 40 different Dutch orthodontists and treated by 14 different OMF surgeons and eight supervised resident OMF surgeons.

### Orthodontic bracket and molar bands

Stainless-steel brackets formed the largest group of type of appliance, being present in 94 (75.8%) patients (Table 1). Ceramic brackets were seen in 30 (24.2%) patients in the anterior region and never distal to the second premolars. Either stainless-steel brackets, bonded tubes or molar bands were used in the posterior area in all patients with ceramic brackets. Molar bands were present in 73 (58.9%) patients. Most of the patients with molar bands only had bands on their first molars and stainless-steel tubes on their second molars (n = 48, 38.7%) (Table 1). The use of bonded tubes alone, without molar bands, was seen in 51 (41.1%) patients.

**Table 1.** General type of bonded orthodontic component.

		Patientsn (%)	Bonded componentsn (%)
Stainless-steel brackets	Twin bracketSelf-ligating bracket Total stainless-steel brackets	44 (35.5)50 (40.3)94 (75.8)	862(26.7) 1064(33.0) 1927(59.7)
Combination ceramic and stainless-steel brackets	Ceramic twin bracket Ceramic self-ligating bracket Total ceramic brackets	23 (18.5)7 (5.6)30 (24.2)	310 (9.6) 115 (3.6) 425 (13.2)
Bonded tubes	On first molar onlyOn second molar onlyOn both molarsTotal bonded tubesTotal brackets	25 (20.1) 48 (38.6) 51 (41.1) 124 (100) 124 (100)	525 (16.3) 2877 (89.1)
Molar bands	On first molar onlyOn second molar onlyOn both molarsTotal molar bands	48 (38.7) 1 (0.8) 24 (19.4) 73 (58.9)	352 (10.9)

### Overview of fixation points

**Figure 1.**
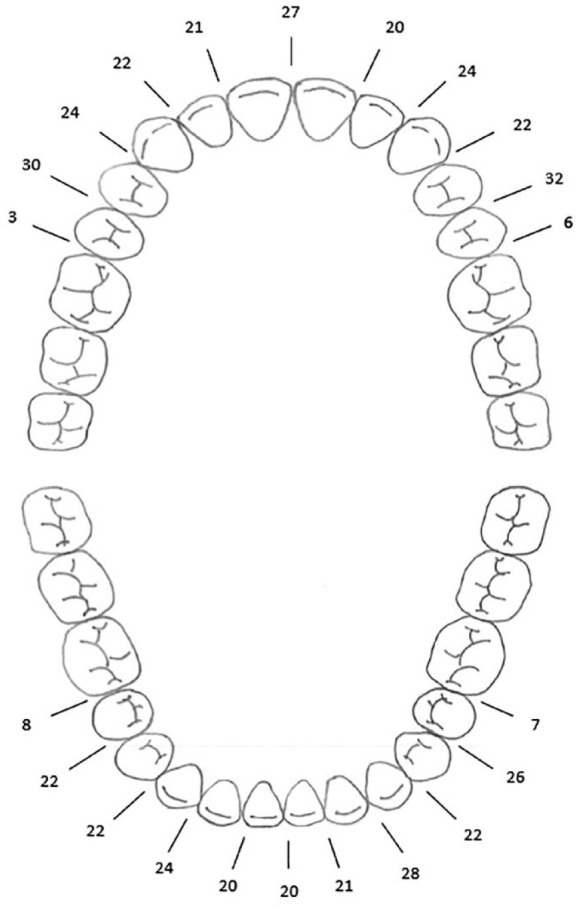
Number of patients with surgical hooks in each inter-dental site.

In 50% of the patients (n = 62), a variable number of stainless-steel hooks were placed on the orthodontic wire, in the range of 2–20 surgical hooks per case (Table 2). In 61 patients, the surgical hooks were crimped to the orthodontic wire and in just one case they were welded to the orthodontic wire. On average, seven surgical hooks were present per case in the anterior region and mesial and distal to the first premolars (Figure 1).

**Table 2.** Overview of fixation points.

		Patients (n (%))	Components (n)	Fixation points (%)
*Hooks on brackets, bonded tubes or molar bands*				
	Stainless-steel twin bracket Stainless-steel self-ligating bracket Ceramic twin bracketCeramic self-ligating bracket Bonded tubes Molar bands Total bonded extensions	44 (35.5) 50 (40.3) 23 (18.5) 7 (5.6) 124 (100) 73 (58.9) 124 (100)	234 361 137 20 525 334 1611	10.2 15.8 6.0 0.9 23.0 14.6 70.5
*Surgical hooks*				
	Crimped Welded Total surgical hooks	61 (49.2) 1 (0.8) 62 (50)	444 8 452	19.4 0.4 19.8
*Kobayashi ligatures*				
	Stainless-steel twin bracket Stainless-steel self-ligating brackets Ceramic twin bracket Ceramic self-ligating bracket Total Kobayashi ligatures	12 (9.7) 3 (2.4) 2 (1.6) 1 (0.8) 18 (14.5)	151 28 18 1 198	6.6 1.2 0.7 0.0 8.7
Powerpins		4 (3.2)	25	1.1
Total fixation points		124	2286	

### Failure rate

A total of 65 components, in 45 (36.2%) patients, were found to have failed. This included 40 (1.3%) brackets and bonds, 2 (0.5%) molar bands, 11 (2.4%) surgical hooks, 6 (3%) Kobayashi hooks and 6 (23.1%) powerpins (Table 3).Two of the molar bands failed, with one band becoming detached while the failure of the other band was described as distortion of the hook. Of the 425 ceramic brackets, 3 (0.7%) failed. In one case, the hook broke off the bracket and in the other two cases, the ceramic bracket became detached at the adhesive-enamel interface. In total, 37 (1.5%) stainless-steel brackets failed. In one of the failed stainless-steel brackets, the hook of the bracket bent. All the other 36 failed brackets became detached.

**Table 3.** Failure rates.

		Failures (n (%))	Patients with failed component (n)	Total frequency of component (n)	Failures per type of component (%)
*Brackets and bonded tubes*					
	Stainless-steel bracket without hook Stainless-steel bracket with hook Bonded tube with hook Ceramic bracket without hooks Ceramic bracket with hooks Total brackets and bonds	9 (13.8) 7 (10.8) 21 (32.2) 2 (3.1) 1 (1.5) 40 (61.6)	4 5 17 2 1 29	1332 595 525 249 157 3157	0.8 0.6 4.0 0.8 0.6 1.3
*Molar bands*					
	With hooks Without hooks Total molar bands	2 (3.0) 0 (0.0) 2 (3.1)	2 0 2	334 18 352	0.6 0.0 0.5
*Surgical hooks*					
	PinchedWeldedTotal surgical hooks	10 (15.4) 1 (1.5) 11 (16.9)	8 1 9	444 8 452	2.3 12.5 2.4
*Kobayashi ligatures*					
	Stainless-steel twin bracket Stainless-steel self-ligating brackets Ceramic twin bracket Ceramic stainless-steel bracket Total Kobayashi ligatures	5 (7.7) 1 (1.5) 0 (0.0) 0 (0.0) 6 (9.2)	2 1 0 0 3	151 28 18 1 198	3.3 3.6 0.0 0.0 3.0
Powerpins	Total powerpins	6 (9.2)	2	26	23.1
Total failures		65	45		

In two cases, an orthodontic molar tube was lost in the surgical wound and in one case, it was lost in the oral cavity, due to detachment of the tube from the most distal molar. All tubes were easily retrieved during the surgery.

Of the 452 surgical hooks, 11 (2.4%) failed (Table 3). They either became detached or loosened from the orthodontic wire causing rotation of the hook around the wire. One of the welded surgical hooks failed, due to bending of the hook when the TIO-MMF was applied for the third time.

In almost one-fifth of all failures, bending of Kobayashi ligatures or powerpins were the cause of failure. In one of these cases, the Kobayashi ligature fully detached from the bracket. The odds for failure of orthodontic auxiliaries was 3.7 times more for Kobayashi ligatures and powerpins compared to all types of brackets (Table 4). The odds for failure of orthodontic auxiliaries, such as surgical hooks surgical stops, Kobayashi ligatures and powerpins was twice that of stainless-steel brackets (OR 2.09, 95% confidence interval [CI] = 1.24–3.32) (Table 5). No statistically significant difference in risk of failure was found between molar bands or ceramic brackets compared to stainless-steel brackets (Table 5).

**Table 4.** Binary logistic regression analyses (*P* ⩽ 0.05) of the risk of failure of molar bands, surgical hooks and Kobayashi ligatures compared to orthodontic brackets.

	N	Failures	B	OR (95% CI)	*P* value
Brackets and bonded tubes	2652	40	Reference		
Molar bands	352	2	−0.98	0.37 (0.09–1.56)	0.18
Surgical hooks	452	11	0.49	1.63 (0.83–3.20)	0.16
Kobayashi ligatures and powerpins	224	12	1.31	3.70 (1.91–7.15)	0.00
Others*	3	0	−17.02	0.00	1.00

* Surgical stop.

CI, confidence interval; OR, odds ratio.

**Table 5.** Binary logistic regression analyses (*P* ⩽ 0.05) of the risk of failure of ceramic brackets and molar bands compared to stainless-steel brackets.

	N	Failures	B	OR (95% CI)	*P* value
Stainless-steel brackets and bonded tubes	2246	37	Reference		
Ceramic brackets	406	3	−0.81	0.44 (0.14–1.45)	0.18
Molar bands	352	2	−1.07	0.34 (0.08–1.43)	0.14
Others*	679	23	0.74	2.09 (1.24–3.32)	0.01

* Surgical hooks, surgical stops, Kobayashi ligatures, powerpins.

CI, confidence interval; OR, odds ratio.

### Time, cause and site of failure

In most cases, the failure occurred during the application of TIO-MMF (70.8% of the patients) (Table 6). In five patients, the failure happened during the removal of the MMF. In eight patients, the time of failure was unknown. The first time of application was most at risk for failure with 46.1% of all failures occurring during this time (Table 6). The most common cause of the failure was due to the fixation of the splint (49 patients, 75.4%). In 6 (9.2%) patients, the failures occurred due to the use of instruments and in 10 (15.4%) patients, the cause was unknown. Most of the failures occurred in the lower jaw (72.3%). The most common site of failure was the most distal molar in the upper or lower jaw (35.4% of all failures) and, in particular, tooth 47 with 10 detached bonded tubes and one bent hook of a molar band.

**Table 6.** Time of failure.

	Failures
Start surgery	6 (9.2)
First time applying MMF	30 (46.1)
First time removal MMF	2 (3.1)
Second time applying MMF	12 (18.5)
Second time removal MMF	0 (0.0)
Third time applying MMF	2 (3.1)
Third time removal MMF	2 (3.1)
Fourth time applying MMF	2 (3.1)
Fourth time removal MMF	1 (1.5)
Unknown moment of failure	8 (12.3)
Total failures at applying MMF	46 (70.8)
Total failures at removal MMF	5 (7.7)
Total failures	65

Values are given as n (%).

MMF, maxillomandibular fixation.

## Discussion

### Summary of the review

Stainless-steel brackets were the most common type of orthodontic bracket present in this study. Ceramic brackets were seen in 25% of the patients and exclusively used in the anterior region. All patients with ceramic brackets had either stainless-steel brackets or molar bands in the posterior region. Molar bands were seen in more than half of all cases and mostly applied only on the first molars. In most patients, bonded tubes were used on the most distal molars. In half of all patients, surgical hooks were seen, varying between 2 and 20 hooks per patient; the most frequent locations were in the anterior region and distal and mesial to the first premolars.

Several previous studies found a greater prevalence of molar bands than bonds (Godoy et al., 2011; Millett et al., 1999; Mizrahi, 1983). This is in contrast with the results of our study, and this may confirm the increased preference for bonded versus banded molars over time (Godoy et al., 2011; Millett et al., 1999; Mizrahi, 1983). Before this study, little was known about the prevalence of stainless-steel brackets, ceramic brackets, surgical hooks and ligature auxiliaries in literature.

### Summery of failure rates

In one-third of all patients, at least one orthodontic bracket or auxiliary failed during surgery. In total, 1.8% of all brackets, bands and auxiliaries failed and could no longer be used as a fixation point for the TIO-MMF. The highest failure rate was seen for modified ligature hooks on the brackets, including Kobayashi ligatures and powerpins. These types of orthodontic auxiliaries tend to distort and bend when high forces are applied on them. Based on this finding, modified ligature hooks are less suitable for fixation of the TIO-MMF. Therefore, could be recommended to fix the splint for the TIO-MMF on surgical hooks, brackets or molar bands with hooks instead.

In contrast to the previous literature describing a higher bond strength of ceramic brackets, no statistical differences were found in the failure rates of ceramic brackets compared to stainless-steel brackets in this study (Jospeh and Rossouw, 1990; Kilponen et al., 2019; Odegaard, 1988; Uysal et al., 2010). In addition, no statistically significant difference in failure rate was found between brackets and molar bands. According to these results, ceramic brackets, stainless-steel brackets and molar bands are all suitable for use in combined orthodontic and surgical cases.

Of all surgical hooks, 2.4% failed, including rotation and sliding along the orthodontic wire, which made them unsuitable for fixation of the TIO-MMF. The failure of crimpable hooks, as well as other types of orthodontic brackets, bands or auxiliaries, may be caused by an incorrect application or the great amount of force loading during application of the TIO-MMF. To facilitate proper fixation of the TIO-MMF, it is recommended that the orthodontist discusses the desired number and location of the surgical hooks with the maxillofacial surgeon.

Almost three-quarters of the failures occurred in the lower jaw. One-third of all failures were noted on the most distal molars in the upper or lower jaw. A standardised bonding system was not seen in this research due to the high heterogeneity of the referring orthodontists. Bonding is a very sensitive procedure and could influence bracket failure. This higher failure rate for the most distal molars could be explained by the possibility of moisture contamination during the bonding procedure in the posterior area, which could decrease the bond strength (Millett et al., 1999). However, in case of a problem or error during the bonding procedure, it could be expected that the bonded tubes would have been detached before surgery due to chewing forces. In addition, the surface area of tubes is less than that for molar bands, so they are more liable to be disloged. The force to debond a tube is probably less than that required to dislodge a molar band. In addition, it could more likely be explained by the fact that in most cases the maxillofacial surgeon started and ended the fixation of the TIO-MMF on the most distal tooth. This might have caused higher forces on the tubes and molar bands on the most distal molars, which could lead to higher failure rates.

According to our data, most failures of the orthodontic brackets, molar bands or auxiliaries occurred while applying the TIO-MMF (70.7%). The first time applying the TIO-MMF appeared to be the most vulnerable moment. This suggests that the number of times the forces, for fixation of the surgical splint, were applied did not have a big influence on the failure probability of the orthodontic bonds, bands or auxiliaries. It demonstrates the possible association between the bond strength and the risk of failure. It suggests that if the bonding strength is low, there might be a higher risk of failure during the first time forces are applied. This is in contrast with the results of the studies by Godoy et al. (2011) and Attishia et al. (2015), which showed a significantly higher risk of failure in two-jaw surgeries and conclude that the more times MMF is applied, the risk of failure of the orthodontic appliances increases.

In three of the participants, the loss of a detached molar tube in the surgical wound or oral cavity was recorded. Because there was no distal stop or bend back at the end of the orthodontic wire, the detached tubes slid off the orthodontic wire. In contrast to the molar tubes, all detached molar bands remained around the molar due to the mechanical retention of the band. Due to the bracket counting for this study, the loss of the bracket was noted before the closing of the surgical wound and end of the surgery. Noticing the lost brackets prevented the loss of the bracket into the surgical wound or aspiration of the bracket or molar tube, when removing the oral tube. This might suggest that the use of molar bands on the most distal molars, crimpable stops or bend backs of the wire distal to the most posterior tube, in combination with active bracket counting at the beginning of the surgery and before closing the surgical wound, may reduce unnoticed loss of an orthodontic bracket, tube or auxiliary into the surgical wound or airway (de Queiroz et al., 2013; Shaeran and Samsudin, 2018).

### Comparison with other work

Since the researchers were present during the orthognathic surgery, a precise review of the types of brackets, molar bands and auxiliaries in situ during surgery and a sound description of the failures, time and cause of failure could be given. This has created a reliable review and evaluation compared to previous studies, which only used postoperative radiographs thus making it hard to determine the exact type of appliances and precise number and time of failures (Attishia et al., 2015; Godoy et al., 2011; Millett et al., 1999; Mizrahi, 1983). For this study, a large number of patients and orthodontic brackets, molar bands and auxiliaries were included. This, in addition to the high number of different maxillofacial surgeons and several different referring orthodontists, means that there was high clinical heterogeneity in this study, which, in turn, may make the results more generalisable.

### Limitations of the project and implications for further study

The number of patients included in this study would have been higher if COVID-19 restrictive measures had not been in place. Increasing the study period and inclusion of more centers for orthognathic surgery could be helpful for further research. In terms of failure rates, it is essential to note which of the hooks and auxiliaries had forces applied on them during the TIO-MMF to generate more reliable data on exact failure rates and cause of failure. As a follow-up study, a case-control study could be proposed to further analyse factors influencing failure.

## Conclusion

Stainless-steel brackets were the most frequently used bracket type in this study. Ceramic brackets were only used in the anterior regions. Molar bands were present in more than half of all cases and largely applied on the first molars only. This confirms the trend for bonded tubes on molars. In one-third of all 124 patients, at least one orthodontic bracket, molar band or auxiliary failed during surgery, which made them unsuitable for the fixation of the TIO-MMF. Three-quarters of all failures occurred in the lower jaw and one-third on the most distal molars. Most failures occurred at the first time of applying fixation forces. This confirms the possible association between bonding strength and risk of failure. We would, therefore, make some recommendations. Due to the high failure rates of Kobayashi ligatures and powerpins, we recommend fixing the TIO-MMF on the bracket hooks, molar bands and/or crimpable hooks. To avoid the loss of a detached bracket or auxiliary in the surgical wound or airway, it is advised that the brackets and surgical hooks are counted at the beginning of the surgery and before closing the surgical wound, with extra attention on the most distal molars. Finally, the use of molar bands on the most distal molars, surgical stops or bend backs of the orthodontic wire distally to the most posteriorly placed brackets or tubes, could prevent the loss of detached brackets or tubes from sliding off the wire during orthognathic surgery.

## Supplemental Material

sj-docx-1-joo-10.1177_14653125231186825 – Supplemental material for A review and evaluation of orthodontic brackets, molar bands and orthodontic auxiliaries during orthognathic surgery: A prospective cohort study Supplemental material, sj-docx-1-joo-10.1177_14653125231186825 for A review and evaluation of orthodontic brackets, molar bands and orthodontic auxiliaries during orthognathic surgery: A prospective cohort study by Romee ME van Ommeren, Tom CT van Riet, Jean-Pierre TF Ho, Ronald EG Jonkman and Alfred G Becking in Journal of Orthodontics
