# Supplementary material for: A review and evaluation of orthodontic brackets, molar bands and orthodontic auxiliaries during orthognathic surgery: A prospective cohort study
Source: J Orthod. 2023 Jul 18;51(1):79–86. doi: 10.1177/14653125231186825 (PMC10924552; doi:10.1177/14653125231186825)
Supplement: sj-docx-1-joo-10.1177_14653125231186825 – Supplemental material for A review and evaluation of orthodontic brackets, molar bands and orthodontic auxiliaries during orthognathic surgery: A prospective cohort study [file sj-docx-1-joo-10.1177_14653125231186825.pdf]

## Appendix 1: Measurement form

*Operator:*
*Orthodontist:*
*Patient male/Female:*
*Is this case usable for research: Yes/no/maybe*

*A review and evaluation of orthodontic brackets, molar bands and orthodontic auxiliaries during orthognathic surgery. A prospective cohort study*

| Tooth number | M1: Type of appliance | Moment of failure | Type of failure | Cause of failure |
|---|---|---|---|---|
| **18** | | | | |
| 18-17 | | | | |
| **17** | | | | |
| 17-16 | | | | |
| **16** | | | | |
| 16-15 | | | | |
| **15** | | | | |
| 15-14 | | | | |
| **14** | | | | |
| 14-13 | | | | |
| **13** | | | | |
| 13-12 | | | | |
| **12** | | | | |
| 12-11 | | | | |
| **11** | | | | |
| 11-21 | | | | |
| **21** | | | | |
| 21-22 | | | | |
| **22** | | | | |
| 22-23 | | | | |
| **23** | | | | |
| 23-24 | | | | |
| **24** | | | | |
| 24-25 | | | | |
| **25** | | | | |
| 25-26 | | | | |
| **26** | | | | |
| 26-27 | | | | |
| **27** | | | | |
| 27-27 | | | | |

*(Continued)*

| Tooth number | M1: Type of appliance | Moment of failure | Type of failure | Cause of failure |
|---|---|---|---|---|
| **28** | | | | |
| **38** | | | | |
| 38-37 | | | | |
| **37** | | | | |
| 37-36 | | | | |
| **36** | | | | |
| 36-35 | | | | |
| **35** | | | | |
| 35-34 | | | | |
| **34** | | | | |
| 34-33 | | | | |
| **33** | | | | |
| 33-32 | | | | |
| **32** | | | | |
| 32-31 | | | | |
| **31** | | | | |
| 31-41 | | | | |
| **41** | | | | |
| 41-42 | | | | |
| **42** | | | | |
| 42-43 | | | | |
| **43** | | | | |
| 43-44 | | | | |
| **44** | | | | |
| 44-45 | | | | |
| **45** | | | | |
| 45-46 | | | | |
| **46** | | | | |
| 46-47 | | | | |
| **47** | | | | |
| 47-48 | | | | |
| **41** | | | | |

Comments:

. . . . . . . . . . . . . . . . . . . . . . . . . . . . . . . . . . . . . . . . . . . . . . . . . . . . . . . . . . . . . . . . . . . . . . . . . . . . . . . . . . . . . . . . . . . . .

. . . . . . . . . . . . . . . . . . . . . . . . . . . . . . . . . . . . . . . . . . . . . . . . . . . . . . . . . . . . . . . . . . . . . . . . . . . . . . . . . . . . . . . . . . . . . .

. . . . . . . . . . . . . . . . . . . . . . . . . . . . . . . . . . . . . . . . . . . . . . . . . . . . . . . . . . . . . . . . . . . . . . . . . . . . . . . . . . . . . . . . . . . . . .

. . . . . . . . . . . . . . . . . . . . . . . . . . . . . . . . . . . . . . . . . . . . . . . . . . . . . . . . . . . . . . . . . . . . . . . . . . . . . . . . . . . . . . . . . . . . . .

| | | | | | |
|---|---|---|---|---|---|
| M1: Figure 8 | Yes/no | Location: | | | |
| M1: Power chain | Yes/no | Location | | | |
| M1: Orthodontic component attached to other than enamel | Ceramic | Gold | Metal | Composite | Glassionomer |
| M1: Mini screw | Yes/no | Location | | | |
| M1: Type of surgery | | | | | |

Comments:

. . . . . . . . . . . . . . . . . . . . . . . . . . . . . . . . . . . . . . . . . . . . . . . . . . . . . . . . . . . . . . . . . . . . . . . . . . . . . . . . .

. . . . . . . . . . . . . . . . . . . . . . . . . . . . . . . . . . . . . . . . . . . . . . . . . . . . . . . . . . . . . . . . . . . . . . . . . . . . . . . . .

. . . . . . . . . . . . . . . . . . . . . . . . . . . . . . . . . . . . . . . . . . . . . . . . . . . . . . . . . . . . . . . . . . . . . . . . . . . . . . . . .

. . . . . . . . . . . . . . . . . . . . . . . . . . . . . . . . . . . . . . . . . . . . . . . . . . . . . . . . . . . . . . . . . . . . . . . . . . . . . . . . .

## Legend

Time 1: (M1): Start of surgery
Time 2: (M2): First time applying TIO-MMF
Time 3: (M3): First time removal TIO-MMF
Time 4: (M4): Second time applying TIO-MMF
Time 5: (M5): Second time removal TIO-MMF
Time 6: (M6): End of surgery
Different (M7): Different: note at comments

- Type of orthodontic component
  1. Twin bracket without hook
  2. Twin bracket with hook
  3. Twin bracket with Kobayashi ligature
  4. Self-ligating bracket without hook
  5. Self-ligating bracket with hook
  6. Self-ligating bracket with Kobayashi ligature
  7. Ceramic twin bracket without hook
  8. Ceramic twin bracket with hook
  9. Ceramic twin bracket with Kobayashi ligature
  10. Ceramic self-ligating bracket without hook
  11. Ceramic self-ligating bracket with hook
  12. Ceramic self-ligating bracket with Kobayashi ligature
  13. Molar band without hook
  14. Molar band with hook
  15. Surgical hook welded on orthodontic wire
  16. Surgical hook crimped on orthodontic wire
  17. Surgical stop welded on orthodontic wire
  18. Surgical stop crimped on orthodontic wire
  19. Multi-loop wire
  20. No appliance
  21. No tooth
  22. Different: note at comments
  23. Power pin

- Type of failure
  1. No failure
  2. Bracket detached, composite on tooth
  3. Bracket detached, composite on bracket
  4. Bracket detached, composite partially on tooth, partially on bracket
  5. Hook breaks
  6. Hook bends
  7. Ligature breaks
  8. Ligature detached
  9. Surgical hook breaks at the lower part (transition point of the orthodontic wire)
  10. Surgical hook detached (rotation/scrolling)
  11. Surgical hook breaks at the upper part (at the curvature)
  12. Surgical stop breaks at the lower part (transition point of the orthodontic wire)
  13. Surgical stop detached (rotation/scrolling)
  14. Molar band detached: still usable for maxillo-mandibular fixation (MMF)
  15. Molar band detached: no longer useable for MMF
  16. Deformation of the orthodontic wire
  17. Different: note at comments

- Cause of failure:
  1. Due to fixation
  2. Due to use of surgical instrument
  3. Different: note reason at comments

(Continued)

Type of orthodontic component.

| | |
|---|---|
| Twin bracket without hook | 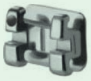 |
| Twin bracket with hook | 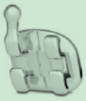 |
| Twin bracket with Kobayashi ligature | 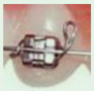 |
| Self-ligating bracket without hook | 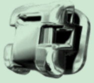 |
| Self-ligating bracket with hook | 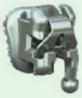 |
| Self-ligating bracket with Kobayashi ligature | 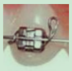 +self-ligating |
| Ceramic twin bracket without hook | 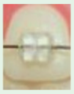 |
| Ceramic twin bracket with hook | 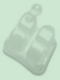 |
| Ceramic twin bracket with Kobayashi ligature | 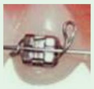 +ceramic |
| Ceramic self-ligating bracket without hook | 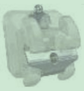 |
| Ceramic self-ligating bracket with hook | 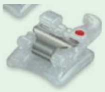 |
| Ceramic self-ligating bracket with Kobayashi ligature | 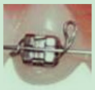 +ceramic +self-ligating |
| Molar band without hook | 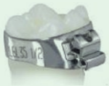 |
| Molar band with hook | 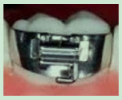 |
| Surgical hook welded on orthodontic wire | 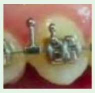 |
| Surgical hook crimped on orthodontic wire | 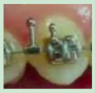 |
| Surgical stop welded on orthodontic wire | 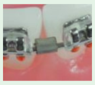 |
| Surgical stop crimped on orthodontic wire | 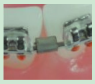 |
| Multi-loop wire | 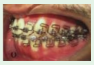 |
| Other | |
| No component on tooth | |
| No tooth | |
| Power pin | 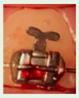 |
